# Comparative Transcriptome Analysis of Human Adipose-Derived Stem Cells Undergoing Osteogenesis in 2D and 3D Culture Conditions

**DOI:** 10.3390/ijms22157939

**Published:** 2021-07-26

**Authors:** Byung-Chul Kim, Kyu Hwan Kwack, Jeewan Chun, Jae-Hyung Lee

**Affiliations:** 1 Department of Dentistry, Graduate School, Kyung Hee University, 26 Kyungheedae-ro, Dongdaemun-gu, Seoul 02447, Korea; xikian@naver.com (B.-C.K.); hahahh@hanmail.net (K.H.K.); jeon385@naver.com (J.C.); 2 Department of Oral Microbiology, School of Dentistry, Kyung Hee University, 26 Kyungheedae-ro, Dongdaemun-gu, Seoul 02447, Korea; 3 Department of Life and Nanopharmaceutical Sciences, Kyung Hee University, 26 Kyungheedae-ro, Dongdaemun-gu, Seoul 02447, Korea

**Keywords:** human adipose-derived stem cells (hADSCs), osteogenic differentiation, three-dimensional (3D) culture, RNA-Seq, bioinformatics

## Abstract

Human adipose-derived stem cells (hADSCs) are types of mesenchymal stem cells (MSCs) that have been used as tissue engineering models for bone, cartilage, muscle, marrow stroma, tendon, fat and other connective tissues. Tissue regeneration materials composed of hADSCs have the potential to play an important role in reconstituting damaged tissue or diseased mesenchymal tissue. In this study, we assessed and investigated the osteogenesis of hADSCs in both two-dimensional (2D) and three-dimensional (3D) culture conditions. We confirmed that the hADSCs successfully differentiated into bone tissues by ARS staining and quantitative RT–PCR. To gain insight into the detailed biological difference between the two culture conditions, we profiled the overall gene expression by analyzing the whole transcriptome sequencing data using various bioinformatic methods. We profiled the overall gene expression through RNA-Seq and further analyzed this using various bioinformatic methods. During differential gene expression testing, significant differences in the gene expressions between hADSCs cultured in 2D and 3D conditions were observed. The genes related to skeletal development, bone development and bone remodeling processes were overexpressed in the 3D culture condition as compared to the 2D culture condition. In summary, our RNA-Seq-based study proves effective in providing new insights that contribute toward achieving a genome-wide understanding of gene regulation in mesenchymal stem cell osteogenic differentiation and bone tissue regeneration within the 3D culture system.

## 1. Introduction

Stem cells are an unspecialized cell type that, if immortalized, can reproduce indefinitely, or can differentiate into one or more types of specialized cells under certain conditions. Therefore, stem cells have been used for the regeneration of lost tissues because they can differentiate into any type of adult tissue. During the last decade, tissue engineering studies using stem cells have been extensively conducted; embryonic stem cells (ESCs) were mainly used in these studies. Since embryonic stem cells have pluripotency, they are thought to be suitable for tissue regeneration research. But the supply of these cells is limited due to the ethical problems involved with the collection of these cells and the technical difficulties involved with harvesting them. This reduces their practical applications for tissue regeneration. Hence, tissue regeneration studies have begun to use adult stem cells instead of ESCs. Although adult stem cells have less ability to differentiate into other tissues as compared to ESCs, they are easier to obtain in greater quantities with fewer ethical and technical complications as compared to embryonic stem cells. 

Human adipose-derived stem cells (hADSCs) have been used to understand the basic biological processes involved in bone differentiation [1]. hADSCs are a type of mesenchymal stem cell that has the ability to differentiate into bone, viscera and skin tissues. In particular, understanding the process of stem cell differentiation into bone cells helps us to figure out the process of bone formation in the early developmental stage, which can aid in the treatment of both bone diseases and bone damage [2,3]. Although previous studies performed using classical two-dimensional (2D) culture conditions (culture plate) successfully showed differentiation of various kinds of stem cells and identified numerous stem cell markers, the products of these studies did not translate into successful clinical applications. There are two reasons why it is difficult to generate artificial tissues for tissue repair applications in 2D culture. First, the shape of regenerated tissue in a 2D culture condition is different from the shape of any actual organ. Though several chemicals or surface-coating molecules, such as lysine and poly-ornithine, can be used to control the cell attachment to the culture plate, it is not easy to control the aggregation of cells themselves on the culture plate [4]. Second, most of the cells in the 2D culture plate are in a mono-layer form rather than a multi-layer form. Thus, under the 2D culture conditions, it is difficult for cells to be surrounded by an extracellular matrix (ECM), which is known to play an important role in maintaining cellular structure and controlling cellular activity. When in vivo, the cells are linked to other cells by ECM released from the cells. These connections are important components of the basic tissue structure [5]. 

To overcome the above limitations, three-dimensional (3D) culture systems were developed to generate cell spheroids in suspension culture [6]. Conventional suspension culture systems cannot produce a constant size of spheroids due to differential cell aggregation ability under varying suspension conditions [7]. In the case of differentiation of stem cells, the irregular sizes of different spheroids can affect the overall interpretation of the analysis results. A microwell plate system can be used as an alternative to control the size of the cultured spheroids [7]. Since the system can control the depth, width and height of the microwell plate, the size of the cultured spheroids can be controlled by manipulating the cell seeded into each well. In addition to spheroid formation, various 3D scaffolds such as collagen sponges, biodegradable molecules, injectable hydrogels, mouse insertions and bioreactors have been applied to stem cell research [8,9]. Collagen encapsulation is a widely used protocol in osteogenic differentiation studies because it can create a similar condition to that of bone tissues where type-I collagen is a main component [10]. Collagen can also effectively stimulate osteogenic differentiation [11]. Recently, the high aspect ratio vessel (HARV) bioreactor system has been used for 3D cultures in addition to conventional suspension cultures. This system has several advantages in the cultivation of cells as described elsewhere [12]. A few studies such as those on stem cell differentiation of cardiac cells [13], intestinal epithelial organoids’ formation on matrigel [14], liver-like tissue formation in a bioreactor [15] and osteogenesis/chondrogenesis from hADSCs in a bioreactor [16] have been presented. Gomez-Roman et al. reported that the 3D culture system for glioblastoma response to drug and radiation therapy was more clinically relevant as compared to the 2D culture system [17]. 

RNA sequencing (RNA-seq) analysis is a powerful tool for efficiently analyzing changes in cells by analyzing the expression levels of all transcriptomes generated in the cell [18]. Meyer et al. analyzed genetic changes of mesenchymal stem cells during osteogenic differentiation using the RNA-Seq and CHIP-Seq techniques. They identified genes associated with osteogenesis that were linked to the RUNX2, C/EBPβ, retinoid X receptor and vitamin D receptor binding sites [19]. To observe the differentiation process of human osteoblasts, Twine et al. measured the whole RNA expression of the differentiated osteoblast from 0 to 12 days. The results showed that the genes related to the formation of the extracellular matrix were significantly overexpressed during bone differentiation [20]. Similarly, it has been shown that long noncoding RNAs have been identified as novel biomarkers for bone differentiation using mouse osteoblast samples [21].

In the present study, we investigated how the hADSCs exhibit different genetic changes under 2D and 3D culture conditions during osteogenic differentiation (Figure S1). Bioinformatic analysis of RNA-Seq data revealed that the gene expression profiles were significantly different between the two different culture conditions.

## 2. Results

### 2.1. hADSCs Spheroid Formation in Microwell

**Figure 1:**
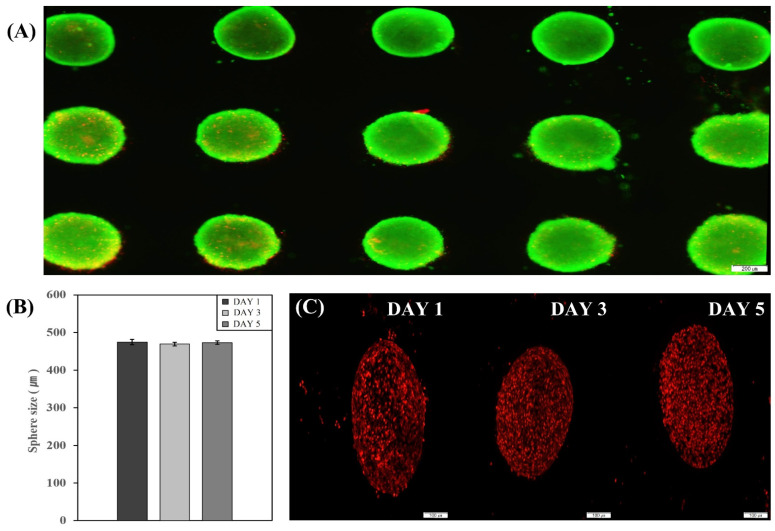
hADSCs spheroids were generated in the non-attachable microwell plate for five days. (**A**) Two-color fluorescence LIVE/DEAD assay. Live cells are shown as a bright green color, while a red-orange color represents dead cells. Scale bar, 200 µm (**B**) The spheroid size of hADSCs at one, three and five days after seeding on microwell plate. (**C**) Annexin V staining assay for identification of cell apoptosis/necrosis at five days after seeding. Scale bar, 100 µm.

To induce the differentiation of hADSCs into bone tissue in the 3D culture condition (in a bioreactor), we first prepared hADSC spheroids. The hADSCs were able to form spheroids through collagen encapsulation in a microwell plate (Figure 1A). We measured the size of spheroids on the first, third and fifth day after seeding. The average sizes of spheroids on the first, third and fifth day after seeding were 475 ± 5 μm, 469 ± 6 μm and 472 ± 5 μm, respectively (Figure 1B). The average size of the spheroid was similar to the size of a spheroid formed in suspension culture conditions in the previous study [22]. To evaluate the spheroid cell viability on the fifth day after seeding, we performed a two-color fluorescence LIVE/DEAD assay that measures intracellular esterase activity and plasma membrane integrity. Although we identified a few dead cells in the spheroids (red dot in Figure 1A), most of the cells were live in the spheroids (green). Furthermore, Annexin V staining assay was performed on the first, third and fifth day after seeding. This was done to assess cell death in the sphere according to the spheroid size (Figure 1C). It has been found that it is difficult to transmit oxygen or nutrients to the center of a larger spheroid as compared to a small spheroid due to limited surface area and since there are no blood vessels in the spheroid. However, even on the fifth day after seeding, the expression of the Annexin V signal was minimal. Hence, we confirmed that substantial cell death did not happen during spheroid formation.

### 2.2. Osteogenic Differentiation of hADSCs in 2D and 3D Culture Conditions

**Figure 2:**
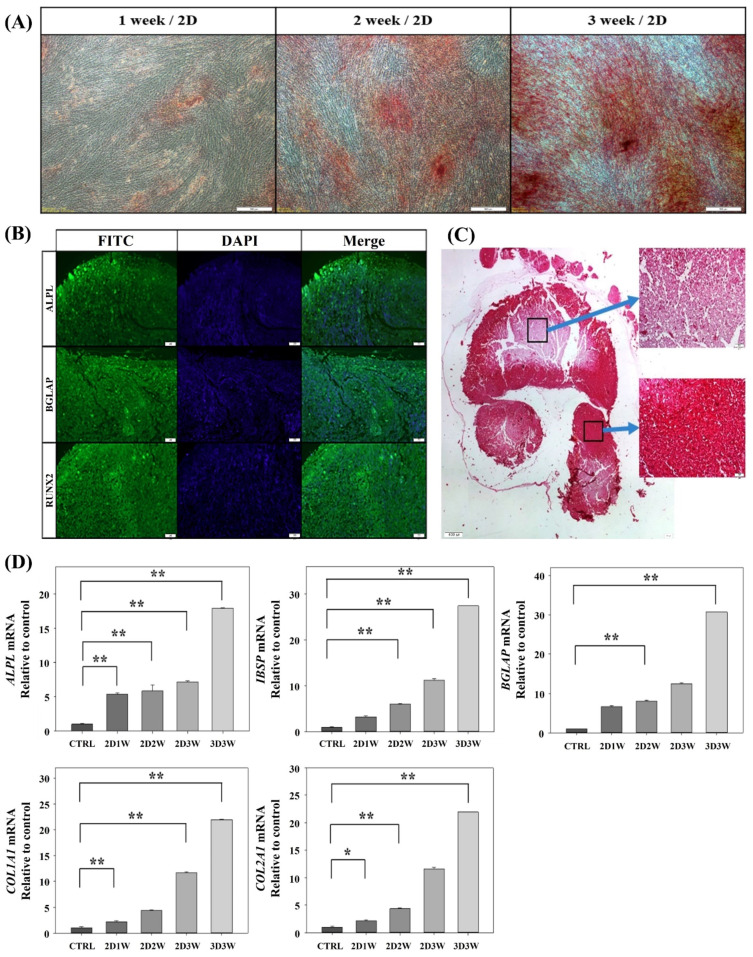
Osteogenic differentiation of hADSCs in 2D and 3D culture conditions. (**A**) Alizarin red staining (ARS) of differentiated hADSCs in 2D culture condition. hADSCs were seeded in a six-well microplate and cultured for three weeks. Every week, the culture samples were fixed and stained. Scale bar, 500 µm (**B**) Immunohistochemical staining was carried out to investigate osteogenic differentiation. Osteogenic differentiation markers were used such as *ALPL*, *BGLAP* and *RUNX2*. Scale bar, 50 µm (**C**) ARS staining of a sliced osteogenic differentiated hADSCs spheroid at three weeks. Scale bar, 400 µm, 50 µm, respectively. (**D**) Quantitative real-time PCR (qRT–PCR) analysis for osteogenic marker expression in 2D and 3D culture conditions. Five genes were used to check for osteogenic differentiation. * *p* < 0.1, ** *p* < 0.05 vs control value.

hADSC is a mesenchymal stem cell line that has the ability to differentiate into bone tissues. It has been shown that hADSCs differentiate into bone cells (osteoblasts) after two to three weeks in osteogenic differentiation culture conditions [23]. To assess and confirm the osteogenic differentiation ability of the hADSCs used in this study, we performed traditional osteogenic differentiation of hADSCs on a culture plate. This represents conventional, 2D culture conditions. The hADSCs were cultured in osteogenic differentiation media for three weeks. Samples were stained with 4% Alizarin Red Solution (ARS) on a weekly basis to check for calcium and phosphate minerals that are associated with bone components. As shown in Figure 2A, the stained area of the cultured samples gradually increased over time. Therefore, we concluded that the hADSCs used in the current study have the ability to differentiate into bone tissues.

For osteogenic differentiation of hADSCs in the 3D culture condition, the spheroids of hADSCs formed five days after seeding were encapsulated by type-I collagen gel. This was done to create an environment representative of being surrounded by natural bone tissue (Figure S2A). This encapsulation environment also prevents physical damage caused by fluid shear forces in the bioreactor [24]. In the 3D culture condition, the size of the cultured spheroid is important due to the limitations of oxygen and glucose diffusion through the spheroid to reach the inner-most cells. The maximum size a spheroid can grow to without necrosis or apoptosis is about 400~500 μm diameter when cultured in normal suspension conditions without special treatment [22,25]. Therefore, we used a HARV-bioreactor to culture larger-sized spheroids. The vessel rotation in the HARV-bioreactor keeps the internal oxygen saturation level of the spheroid high. This prevents necrosis of the internal cells in the spheroids [26] (Figure S2B). Immunohistochemical staining was performed after three weeks using known bone differentiation markers such as *ALPL*, *BGLAP* and *RUNX2* [27]. As shown in Figure 2B, we confirmed that all three markers were positive in the spheroid sample, indicating osteogenic differentiation. Histological analysis was also performed to evaluate the progress of osteogenesis inside the spheroid. The spheroid was dissected and stained with ARS. Although the degree of staining of the cells around the spheroid center was weak, most cells in the spheroid were well stained (Figure 2C).

Next, we performed qRT–PCR to compare the osteogenic differentiation status of hADSCs depending on the culture conditions and time. Five generic osteogenic differentiation-related genes—*BGLAP*, *ALPL*, *IBPS*, *COL1A1* and *COL2A1* [28,29,30,31]—were tested. As expected, the expression levels of the markers increased over time in the 2D culture condition, and the expression patterns of the five markers were similar to each other. Interestingly, the expression levels of all five markers in the 3D culture condition at three weeks were greater than the ones in the 2D culture condition at the same culture time (Figure 2D).

### 2.3. Gene Expression Profiles during Osteogenic Differentiation of hADSCs

**Figure 3:**
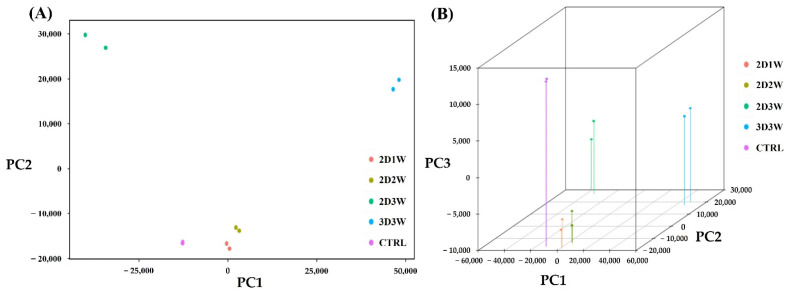
Principal component analysis of osteogenic differentiated samples in 2D condition (one, two and three weeks) and 3D condition (three weeks). (**A**) Two-dimensional scatter plot for principal components. (**B**) Three-dimensional scatter plot for three principal components.

To delineate the detailed molecular mechanisms during osteogenic differentiation of hADSCs, we conducted whole transcriptome sequencing. We extracted total RNA from samples at each time point (one, two and three weeks for 2D (2D1W, 2D2W and 2D3W), three weeks’ differentiation for 3D (3D3W)). Additionally, an undifferentiated control sample was sequenced (undifferentiated hADSCs; CTRL). In each sample, two biological replicates and a total of approximately 310 million (~30 million pairs of reads per sample) pairs of reads (2 × 101 bp) were obtained. The raw reads were aligned to the hg38 human reference genome and approximately 273 million pairs of reads were uniquely mapped and properly paired (Table S1). Based on the Ensembl release 88 annotations, a total of 31,104 genes had at least one read. Principal component analysis (PCA) showed that 93.3% of the variation in gene expression was caused by the first two principal components (PC1: 62.2% and PC2: 31.1%). Interestingly, the first PC isolated the data according to the differentiation status (CTRL samples were close to the 2D1W and 2D2W samples). The second PC further separated the differentiated samples based upon the culture conditions, i.e., 2D vs. 3D at three weeks’ differentiation (Figure 3).

### 2.4. Identification of Differentially Expressed Genes during Osteogenic Differentiation

**Figure 4:**
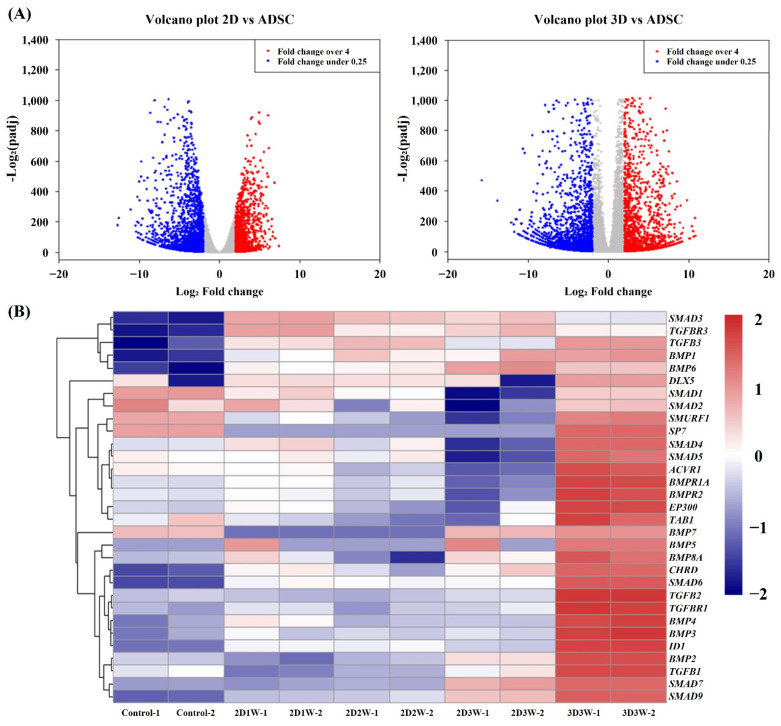
Differentially expressed genes after the differentiation of hADSCs. (**A**) Volcano plots for differential gene expression. Comparison between undifferentiated sample (ADSC) and differentiated sample in 2D and 3D culture conditions. The x and y axes represent the magnitude of fold changes (log2 transformed) and the adjusted *p*-value (−log2) by Benjamini-Hochberg correction, respectively. (**B**) A heatmap of the 31 genes associated with the TGF-β signaling pathway. The gene expression of each gene was normalized using TPM and converted into a z-score.

Both the 2D and 3D cultured hADSCs (2D3W and 3D3W) were compared with undifferentiated hADSCs (CTRL) to identify differential gene expression. The differential gene expression tests were performed by the DEseq2 package in R [32]. Differentially expressed genes were defined by at least a four-fold change with 5% FDR between samples. In the 2D culture condition, a total of 3503 genes were differentially expressed (upregulated: 2347 and downregulated: 1156). Similarly, a total of 3894 genes were differentially expressed (upregulated: 2368 and downregulated: 1526) in the 3D culture condition (Figure 4A). We validated the gene expression changes by randomly selecting 11 genes using quantitative real-time RT–PCR of the same hADSCs. The validations demonstrated that the results from RNA-Seq and real-time PCR were highly consistent. Pearson’s correlation coefficients were 0.85 (*p* = 5.618 × 10^−6^ and 0.95 (*p* = 1.025 × 10^−4^) in the 2D and 3D culture conditions, respectively (Figure S3).

To explore the biological functions of the differentially expressed genes, we performed functional annotation analysis using Gene Ontology (GO) terms and KEGG pathway information. The results showed that many functions related to cell differentiation, development and the extracellular matrix such as “cell adhesion molecules”, “skeletal system development”, and “osteoblast differentiation” were enriched in the upregulated genes in both culture conditions. On the other hand, functions related to the cell cycle and cell division such as “mitosis”, “chromosome segregation” and “DNA replication” were enriched in downregulated genes. More specifically, in the 3D culture condition, there were more genes related to the bone tissue functions such as “skeletal system development”, “cartilage development” and “odontogenesis” compared to the upregulated genes in the 2D culture condition. Since it has been shown that the TGF-β signaling pathway is strongly associated with osteogenic differentiation [33], we chose 31 genes related to the TGF-β signaling pathway and checked the expression levels of those genes. As shown in Figure 4B most of the genes were highly expressed in the 3D culture conditions.

### 2.5. Identification of Molecular Signature Specific to Samples in 3D Culture Condition

**Figure 5:**
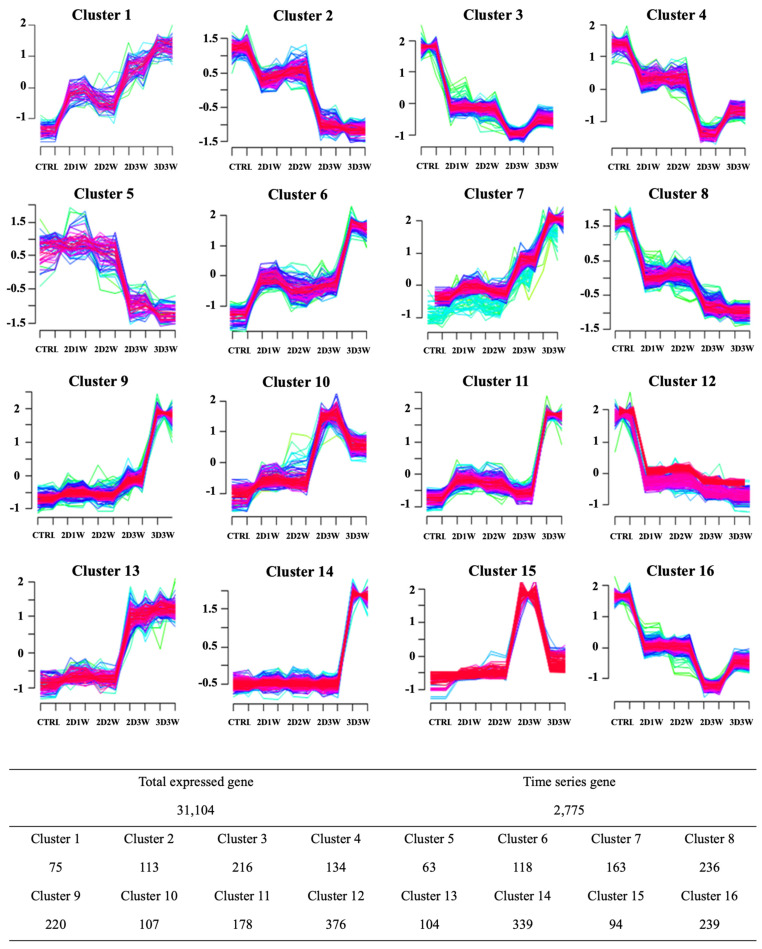
Clustering analysis of time-series genes during osteoblast differentiation using Mfuzz. The x-axis represents each sample. The table below the cluster figures shows the number of genes in each cluster.

As described in the previous sections, many genes were differentially expressed during the osteogenic differentiation process. To carefully characterize the temporal gene expression changes, we applied time course analysis to the whole of the transcriptome expression data during the differentiation. Two methods, DESeq and edgeR, were used to detect the genes that were differentially expressed over the time course. In total, 2775 genes were significantly differentially expressed. We called those 2775 genes differentially expressed time series genes, which were further grouped according to their normalized gene expression patterns by the fuzzy c-means algorithm implemented in the R Mfuzz package [34]. We obtained 16 different clustered groups and each group had 94 ~ 376 genes (Figure 5). We focused on the three clusters (clusters 9, 11 and 14) with highly expressed genes only in the 3D cluster condition in order to determine how the 3D culture conditions differently affected gene expression and biological functions during osteogenic differentiation. The three clusters included 737 genes, which were enriched with functions associated with “extracellular matrix organization”, “skeletal system development”, “osteoblast differentiation”, “BMP signaling pathway”, “collagen fibril organization”, “embryonic skeletal system morphogenesis” and “odontogenesis of dentin-containing tooth” (Table 1).

**Figure 6:**
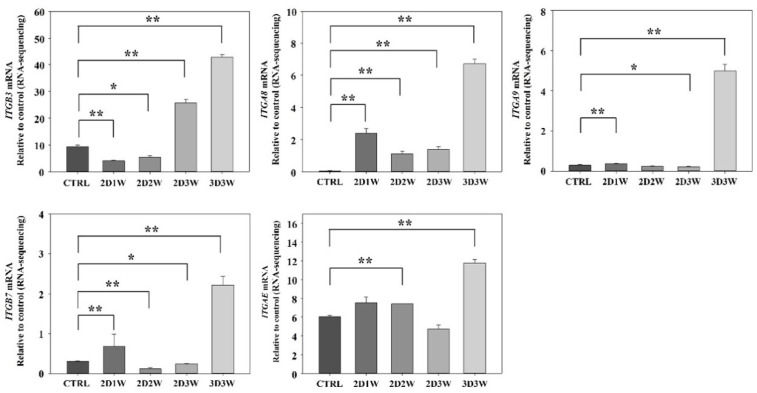
Five integrin gene expressions in RNA-Seq data. Integrin genes known to be related to osteogenic differentiation were selected and are described in the discussion section. * *p* < 0.1, ** *p* < 0.05 vs control value.

We examined the level of expression of integrin, which plays an important role in connecting cells to cells. It is involved in cell signaling by a combination of several α units and β units. This protein regulates several cellular processes such as proliferation, apoptosis and differentiation [35]. We checked the whole transcriptome expression data and found that five integrin genes—*ITGB3*, *ITGA8*, *ITGB7*, *ITGAE* and *ITGA9*—had significantly higher levels of expression in the 3D culture condition sample as compared to the samples from the 2D culture condition (Figure 6).

## 3. Discussion

Due to the ability of adult stem cells to differentiate into tissues or organs, tissue regeneration using stem cells has been extensively studied for clinical application. Various methods such as simple cell bundles, metal-based implants, biodegradable sponges, ECM hydrogels and powdered bone have been used to generate in vitro 3D bone tissues. Numerous scaffolds containing stem cells capable of differentiating into bones have been used to create artificial bones similar to bone tissue [36,37,38]. We employed three different strategies to generate in vitro 3D bone tissue by differentiating hADSCs. First, the microwell was used to control the size of the spheroid in the 3D culture condition. Because the microwell is comprised of agarose, which the cells cannot attach, we can control the size of the spheroid by adjusting the number of cells injected [7]. Second, the bioreactor for the 3D culture was used to generate larger tissue components because it is easy to control the size of the cultured tissue [26]. Lastly, we used Type-I collagen gel to encapsulate the cultured spheroid. Type-I collagen is the basic material that constitutes the bone and promotes bone differentiation of stem cells. It also protects the spheroid from breakages caused by rotation of the media in the bioreactor [24]. Dozio et al. [39] reported that the osteogenic induction of human mesenchymal stem cells in a 3D hybrid scaffold culture was more effective as compared to osteogenic induction in 2D standard culture systems. They did this using only a handful of differentiation markers. Our present study highlights the power of 3D culture systems in osteogenic differentiation of hADSCs based on whole transcriptome profiles. By monitoring and comparing the gene expression profiles of the differentiated hADSCs between the 2D culture condition and the 3D culture condition, we were able to assess the biological benefit of the 3D system. These results showed that the gene expression patterns were significantly different between the 2D culture condition and the 3D culture condition.

hADSCs are a kind of mesenchymal stem cells that have osteogenic differentiation ability. In bone differentiation, hADSCs differentiate into pre-osteoblasts, and then the pre-osteoblasts differentiate into osteoblasts and osteocytes. To check the phenotype of the osteogenic differentiation of hADSCs, we stained the differentiated cultured cells using ARS in both conditions and confirmed that the hADSCs successfully differentiated. Although the degree of the ARS staining was weak in the cells near the spheroid center for the 3D culture condition, we could not find any dead cells or cavitation in the center of the spheroid. Previously, various genes have been identified as genetic markers for the initial stage of the bone differentiation process of the ADSCs. *BGLAP* (bone gamma-carboxyglutamate protein) is a hormone secreted from the osteoblast. It acts on itself and stimulates osteogenic differentiation from osteoblast to bone tissue. Also, it plays various biological and physiological roles such as bone formation, bone mineralization and calcium ion homeostasis [29]. *ALPL* (alkaline phosphatase) is a tissue-specific enzyme expressed in intestinal, placental, placental-like and liver/bone/kidney tissues. In the early stages of bone development, it plays a role in initiating bone mineralization on the cell surface and in the matrix vesicles [30,31]. *COL1A1* and *COL2A1* are collagen genes. They are also known as early markers of osteoblast differentiation [10]. *IBPS* is known to be expressed in bone, dentin, cementum and calcified cartilage [28,40]. Therefore, we selected those five genes to evaluate osteogenic differentiation by measuring the gene expression levels. The magnitude of the gene expression levels of the five marker genes in the 3D culture condition was more than twice as big as compared to the magnitude in the 2D culture conditions. Similarly, previous studies demonstrated increased osteogenic differentiation of mesenchymal stem cells in 3D vs. 2D when gelatin or a mix of gelatin and alginate were used as a 3D scaffold [41]. Hence, at the molecular level, the genes associated with bone morphogenesis are more highly expressed in the 3D culture condition as compared to the 2D culture condition.

The RNA-Seq analysis results showed that various functional genes associated with bone development such as “skeletal development function”, “BMP signaling” and “osteoblast differentiation” were enriched for the overexpressed gene set cultured in the 3D condition. Bioinformatic clustering analysis with temporal gene expression profilometry indicated higher expression for genes with functions related to the extracellular matrix, skeletal development, collagen fibril organization, BMP signaling pathways and osteoblast differentiation for the 3D culture condition. Previously, it was shown that the BMP signaling pathway plays a fundamental role in bone formation [5]. TGF-β pathways linked with the BMP signaling pathway are involved in the regulation of bone organogenesis by activation of various kinases [42,43]. We picked 31 genes related to osteoblast differentiation and osteogenesis from the TGF-β/BMP signaling pathway and investigated the expression levels of these in the samples. The expression profiles showed how the 31 genes, including BMP2, were expressed relative to each other under different culture conditions. We observed that most of the genes were highly expressed in the samples of the 3D culture condition as compared to others. Recently, Lin et al. showed that BMP2-engineered human bone marrow-derived stem cells in a hydrogel scaffold promoted bone formation in vitro and in vivo [44]. Likewise, the other molecular markers associated with the TGF-β/BMP signaling pathway identified in our study could be used as potential factors to promote bone formation and regeneration.

Cells communicate with each other by interacting through ECM, and ECM is known to play an important role in bone formation. For these reasons, ECM has attracted much attention as a material for applications in bone tissue engineering [45]. Integrin is a transmembrane heterodimer receptor composed of non-covalently-linked alpha and beta subunits. It binds to extracellular matrix components such as collagen, laminin, pyronectin, bitronine, thrombospondin, osteopontin and tenestin. In this way, diverse cellular processes such as cell attachment, spreading, migration and differentiation are regulated by the combination of different integrin units [46]. For example, integrin α2β1 is known to affect bone differentiation [5]. We reasoned that the expression patterns of the integrin family associated with osteogenesis could be different in different culture conditions. We found that the expression levels of five integrins, comprising two alpha units and three beta units (*ITGB3*, *ITGA8*, *ITGA9*, *ITGAE* and *ITGB7*), under the 3D culture condition were higher than the levels under other conditions. The integrins *ITGB3*, *ITGA8* and *ITGA9* are located outside of the cell membrane and are involved in promoting osteogenesis or initiating osteogenesis by binding to other cells or ECMs. *IGTAE* and *ITGB7* are mainly present on the T cell and are known to be involved in T cell regulation [47]. However, these have not been reported to have functional roles associated with osteogenic differentiation. Therefore, further studies investigating the precise molecular mechanisms associated with integrin-mediated cell-matrix interactions are required to fully understand the role of these integrin genes during bone formation and regeneration. 

There are several limitations to the current study. Because the findings are mainly based on bioinformatic observations at the RNA level, the functional relevance and detailed mechanisms would need to be further investigated experimentally at the cellular and molecular levels. Additionally, more precise culture systems that mimic in vivo environments, such as combinations of various 3D scaffolds and organoids, could give us more realistic clues as to how to develop therapeutic applications for bone regeneration.

## 4. Materials and Methods 

### 4.1. Cell Culture and Osteogenic Differentiation 

Human adipose-derived stem cells (hADSCs) were purchased from Lonza Inc. (Walkersville, MD, USA). The cells were maintained in Dulbecco’s Modification of Eagle’s Medium/Ham’s F-12 50/50 Mix (DMEM/F12; catalog number: 10-090-CV, Corning Life Sciences, NY, USA) supplemented with 10% (*v*/*v*) fetal bovine serum (FBS; catalog number: 35-015-CV, Corning), 1% penicillin-streptomycin (Pen/Strep; catalog number: 15140122, Thermo Fisher Scientific, Waltham, MA, USA), 10 ng/mL recombinant human HB-EGF (EGF; catalog number: 100-47, PeproTech, Rocky Hill, NJ, USA) and 2 ng/mL recombinant human FGF-acidic (bFGF; catalog number: 100-17A, PeproTech, Rocky Hill, NJ, USA) at 37 °C in a humidified atmosphere containing 5% CO_2_. In order to induce osteogenic differentiation in the 2D culture condition, hADSCs suspension (1 ×$\;10^{5}$ cells/six-well) was inoculated in a six-well plate at 1 mL/well. After 1 h, the cells were attached to the plate and incubated in an osteogenic medium composed of high glucose (DMEM; catalog number: 10-013-CV, Corning), 10% (*v*/*v*) FBS, 1% Pen/Strep, 10 mM β-glycerol-phosphate (catalog number: 50020, Sigma-Aldrich; Merck KGaA, Darmastadt, Germany), 100 ng/mL dexamethasone (catalog number: D2915, Sigma-Aldrich; Merck KGaA) and 50 μg/mL ascorbic acid (catalog number: A4403, Sigma-Aldrich; Merck KGaA). The osteogenic medium was refreshed every two days.

### 4.2. Concave Microwell Preparation

Next, 1% *w*/*v* agarose (catalog number: A9539, Sigma-Aldrich, St. Louis, MO, USA; Merck KGaA) with Dulbecco’s phosphate-buffered saline (DPBS; catalog number: 21-031-CV, Corning) was melted in a microwave oven for 2~3 min until the solution was clear. While the solution was hot, 500 μL of melted solution was gently moved to the microwell mold (3D Petri Dish^®^—MicroTissues, catalog number: Z764019, Sigma, St. Louis, MO, USA) without air bubbles forming. After cooling for 3 min at room temperature, the microwells were carefully separated from the mold. The microwell formation was monitored by microscope. In this study, we chose a mold with a well size of 800 μm diameter.

### 4.3. Generation of hADSCs Spheroids and Osteogenic Differentiation in 3D Culture Condition

To generate hADSCs spheroids and induce osteogenic differentiation in the three-dimensional (3D) culture condition, the hADSC suspensions (approximately 1 × $10^{6}$ cells/150 μL) were moved into a concave microwell with a diameter of 800 µm, then incubated in a humidified atmosphere with 5% CO_2_ at 37 °C. After 1 h, each microwell was washed with DPBS carefully to remove undocked hADSCs. Initially, the cells were cultured with a normal growth medium and the volume of the medium was increased by 20% each day. After five days, hADSCs spheroids were encapsulated in 0.1% type-I collagen (Collagen I, Rat Tail, catalog number: A1048301, Thermo Fisher Scientific, Waltham, MA, USA) with the hanging-drop method. The diameter of each gel drop was about 40 μm, and about 10~15 spheroids were in each gel drop. After incubating the gel drops at 37 °C in a humidified atmosphere containing 5% CO_2_ for 1 h, the gel drops were ripped off the gel, removed from the plate and transferred into a 50 mL high-aspect rotating vessel-type bioreactor (disposal vessels; Synthecon, Houston, TX, USA). To induce osteogenic differentiation, the gel drops were cultivated in the bioreactor while rotating at a speed of 30× *g* rpm for 21 days. The osteogenic differentiation medium was changed every two days.

### 4.4. Quantitative Real-Time Polymerase Chain Reaction (qRT–PCR)

Total RNAs were extracted from 2D- and 3D-cultered hADSCs using TRIzol™ reagent (catalog number: 15596026, Thermo Fisher Scientific, Waltham, MA, USA). In the 2D culture condition, cultured cells were washed with DPBS to remove extra proteins from FBS and trypsinized with TrypLE™ Express (catalog number: 12605036, Thermo Fisher Scientific, Waltham, MA, USA) at 37 °C for 3 min. To neutralize TrypLE™ Express, the cells were resuspended in growth media and centrifuged at 1000× *g* rpm for 3 min. After removing the supernatant, Trizol reagent was added to each sample. In the case of 3D spheroids, the cultured spheroids were frozen with liquid nitrogen and Trizol reagent was added after crushing the frozen samples. After measuring the concentration of extracted RNA using NanoDrop™ 2000 (Thermo Fisher Scientific, Waltham, MA, USA), cDNA was synthesized with AccuPower^®^ CycleScript RT PreMix, dT20 (catalog number: K-2044, Bioneer, Daejoen, Korea). Analysis of mRNA expression was carried out using an AccuPower^®^ 2X Greenstar qPCR Master Mix (catalog number: K-6253, Bioneer) with Rotor-Gene Q (Qiazen, Hilden, Germany) by following the manufacture’s protocols. The primers for qRT–PCR were designed with primer 3; the primer sequences are listed in Table S2.

### 4.5. Histological Analysis of 3D Spheroid

The cultured 3D spheroids were fixed in 3.9% paraformaldehyde solution for two days at 4 °C. After fixation, the samples were dehydrated at room temperature with 75% ethanol; the ethanol solution was changed every hour while increasing the ethanol concentration by 5% until it reached 100%. Also, the samples were transferred to 100% xylene solution and embedded in a paraffin solution at 60 °C for 1 day when the samples were translucent. The paraffin-embedded samples were hardened on the chiller for paraffin sectioning. The solidified paraffin block was sectioned at a 7 μm thickness.

### 4.6. Alizarin Red Staining

Alizarin red staining was used to assess the osteogenic differentiation of hADSCs. In the 2D culture condition, the cultured cells were fixed using 3.9% paraformaldehyde solution for 1 h at room temperature and rinsed with DPBS once. The cells were stained with 4% Alizarin Red solution (ARS) for 1 h with light blocking and rinsed five times with DPBS to remove non-specific stained cells. In the case of the 3D culture condition, the sample slides were soaked in 100% xylene solution for 1 h. Afterward, the samples were moved to the 100% ethanol solution. The concentration of ethanol solution was gradually reduced by 5% per hour until the ethanol concentration reached 70%. The samples were washed with tap water for 1 h and were stained with 4% ARS solution for 1 h with light blocking. They were then washed with DPBS once and rewashed with tap water until the water running away did not turn red.

### 4.7. RNA Sequencing (RNA-Seq) and Read Mapping

To perform whole transcriptome sequencing, 1 μg of total extracted RNA was used. A sequencing library was generated using a Truseq Stranded mRNA LT Sample Prep Kit. Around 300 bp fragments were isolated using gel electrophoresis, amplified by PCR and sequenced on the Illumina HiSeq 2500 in the paired-end sequencing mode (2 × 101 bp reads). The quality of the generated raw sequencing reads was carefully inspected. The qualified raw reads were aligned to the hg38 genome using STAR (Ver. 2.5.2b) [48] with the default option. After mapping, the uniquely mapped reads were used for further studies.

### 4.8. Principal Component Analysis

To evaluate the expression levels of genes, the Transcripts Per Million (TPM) measurement unit was used [49] with the gene annotation information downloaded from Ensembl (release 88) biomart (http://www.ensembl.org/, 1 March 2017). Principal component analysis (PCA) was performed in the R prcomp function with the normalized TPM value of each gene.

### 4.9. Differential Gene Expression (DGE) Analysis and Functional Annotations for the Differentially Expressed Genes

The DESeq2 R package (version 1.10.1) [32] was used to identify differentially expressed genes among samples in different conditions and differentiation stages. The false discovery rate (FDR) was controlled by adjusting the *p*-values using the Benjamini–Hochberg algorithm. Thresholds were used to define differentially expressed genes between samples: (1) the adjusted *p*-value is equal to or less than 0.05, and (2) the fold change between groups is greater than 4. To visualize the differentially expressed genes, volcano plots were generated by the ggplot2 packaged implemented in R. Gene Ontology (GO) terms of each gene were downloaded from Ensembl (release 88) biomart (http://www.ensembl.org/, 1 March 2017) and KEGG pathway information was obtained from the KEGG PATHWAY database (http://www.genome.jp/kegg/pathway.html, 1 March 2017). Functional annotations on the gene sets using GO terms and KEGG pathways were performed similarly to as described in Lee et al. [50].

### 4.10. Time-Series Analysis of Differential Gene Expression

R packages DESeq [32] and EdgeR [34] were used to identify genes that were differentially expressed across the differentiation time-period to a significant range, designated time-series genes. We selected time-series genes that displayed significant differential expression with FDR <5% in both DESeq and EdgeR. The identified time-series genes were clustered using the R package Mfuzz [34] that performs soft clustering based on the fuzzy c-means algorithm. Average TPM values (duplicates at each time-point) of individual genes were employed as input values for Mfuzz clustering. The number of clusters was set to 16 and the fuzzifier coefficient, M, to 1.5.

### 4.11. RNA Sequencing Validation by qRT–PCR

A total of 11 genes (ECAD, NCAD, SNAI1, VIM, E2F1, CCND1, CCNE1, MKI67, COL1A1, ALPL, IBSP, ACTB) were used for qRT–PCR. One Step SYBR^®^ PrimeScript™ was used and the total number of cycles was set to 34 cycles. The sequences of the PCR primers are listed in Table S3.

## 5. Conclusions

In the current study, we demonstrated comprehensive transcriptome expression profiles during osteogenic differentiation of hADSCs under different culture conditions, and how the 3D culture environment affects the osteogenic differentiation process at the molecular level. Notably, the findings of the current study could have therapeutic applications, such as in designing molecular factors for various bone regeneration scaffolds. Together, the presented outcomes provide significant indications to guide future research to produce comprehensive insights into bones’ differentiation and regeneration mechanisms. This understanding can then aid in developing strategies for bone tissue regeneration and transplantation.

## Figures and Tables

**Table 1:** Functional annotations for the differentially expressed genes using GO terms.

GO Term	GO Term ID	Gene Count	*p*-Value
extracellular matrix organization	GO:0030198	23	<0.0001
skeletal system development	GO:0001501	17	<0.0001
angiogenesis	GO:0001525	16	0.0003
osteoblast differentiation	GO:0001649	11	<0.0001
BMP signaling pathway	GO:0030509	10	<0.0001
embryonic limb morphogenesis	GO:0030326	10	<0.0001
cartilage development	GO:0051216	10	<0.0001
collagen fibril organization	GO:0030199	9	<0.0001
embryonic skeletal system morphogenesis	GO:0048704	9	<0.0001
odontogenesis of dentin-containing tooth	GO:0042475	9	0.0001

## Data Availability

Not applicable.

## Supplementary Materials

Supplementary material can be found at https://www.mdpi.com/article/10.3390/ijms22157939/s1.
