# Supplementary material for: Comparative Transcriptome Analysis of Human Adipose-Derived Stem Cells Undergoing Osteogenesis in 2D and 3D Culture Conditions
Source: Int J Mol Sci. 2021 Jul 26;22(15):7939. doi: 10.3390/ijms22157939 (PMC8347556; doi:10.3390/ijms22157939)
Supplement: Supplementary file 1 [file ijms-22-07939-s001.zip › ijms-1300716-supplementary.pdf]

Table S1. RNA-seq read mapping summary

Sample name	Total raw reads	Uniquely mapped reads	Mapping rate
CTRL-1	61,379,432	54,804,208	89.29%
CTRL--2	63,426,692	55,891,690	88.12%
2D1W-1	61,697,286	54,216,198	87.87%
2D1W-2	63,548,852	56,065,164	88.22%
2D2W-1	61,697,286	56,185,834	91.07%
2D2W-2	61,480,392	53,737,420	87.41%
2D3W-1	61,299,496	50,092,748	81.72%
2D3W-2	61,545,742	52,020,428	84.52%
3D3W-1	63,036,064	57,851,746	91.78%
3D3W-2	61,269,406	56,145,472	91.64%

Table S2. A list of primers for qRT-PCR to check the osteogenic differentiation

Gene	Primer	Sequence
IBSP	forward	GACTCTGAGGCTGAGAATACCAC
IBSP	reverse	CTTCTTGGGAAGCTGGATTG
COL1A1	forward	CACCAGGGGATCCTTTCTC
COL1A1	reverse	GGTCCTGCTGGCAAAGAAG
COL2A1	forward	AGGGACTTGAGTGTGGCATC
COL2A1	reverse	CTTAGGCCCGAGAGAGAAGG
ALPL	forward	AACACCACCCAGGGGAAC
ALPL	reverse	TGAGTACCAGTCCCGGTCAG
BGLAP	forward	GCAGCGAGGTAGTGAAGAGAC
BGLAP	reverse	TCAGCCAACTCGTCACAGTC
ACTB	forward	AGCACTGTGTTGGCGTACAG
ACTB	reverse	AGAGCTACGAGCTGCCTGAC

Table S3. A list of primers for qRT-PCR for validation of gene expression levels estimated by RNA-Seq

Gene	Primer	Sequence
ECAD	forward	AGGCCAAGCAGCAGTACATT
ECAD	reverse	CATTCACATCCAGCACATCC
NCAD	forward	AGTCGAACAGCAGCTCTGAA
NCAD	reverse	TGGCTTCTCTTTGGCTTCTG
SNAI1	forward	CCAATGCTCATCTGGGACTC
SNAI1	reverse	CCTCATCTGACAGGGAGGTC
VIM	forward	GAGAACTTTGCCGTTGAAGC
VIM	reverse	TGGTATTCACGAAGGTGACG
E2F1	forward	TGGTGGTGGTGACACTATGG
E2F1	reverse	ATGTTTTCCTGTGCCCTGAG
CCND1	forward	TGAACTACCTGGACCGCTTC
CCND1	reverse	GGGGATGGTCTCCTTCATC
CCNE1	forward	ACCCCTGCTCCCTGATCC
CCNE1	reverse	GGGAGCCTCTGGATGGTG
MKI67	forward	TCTCCTGTTGGCTCTGTGTG
MKI67	reverse	TACCCCTGGTGAAAGTGGAC
COL1A1	forward	CACCAGGGGATCCTTTCTC
COL1A1	reverse	GGTCCTGCTGGCAAAGAAG
ALPL	forward	AACACCACCCAGGGGAAC
ALPL	reverse	TGAGTACCAGTCCCGGTCAG
IBSP	forward	GACTCTGAGGCTGAGAATACCAC
IBSP	reverse	CTTCTTGGGAAGCTGGATTG
ACTB	forward	AGGTGTGGTGCCAGATTTTC
ACTB	reverse	GAGGCCCAGAGCAAGAGAG

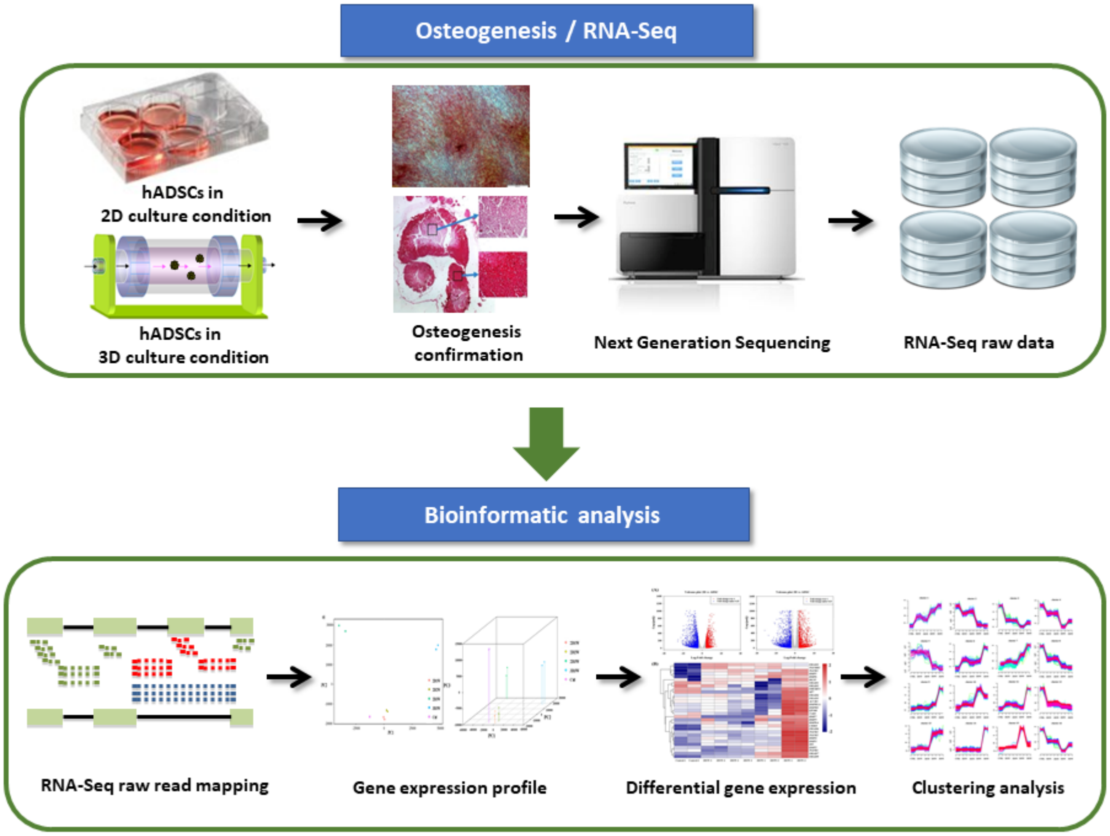

Figure S1. A flowchart of experimental and computational analysis of the study.

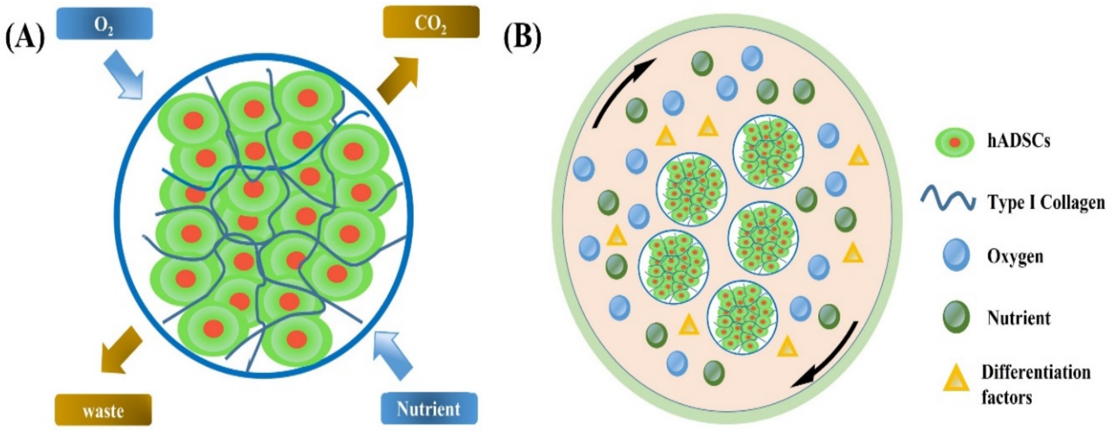

Figure S2. Schematic diagrams of collagen encapsulation and bioreactor (A) hADSCs spheres were encapsulated by type Ⅰ collagen gel. Oxygen, nutrients can be transferred into the spheroids through the gel, and CO2 and wastes can diffuse out of gel (B) Collagen gel beads are freely floating in the media.

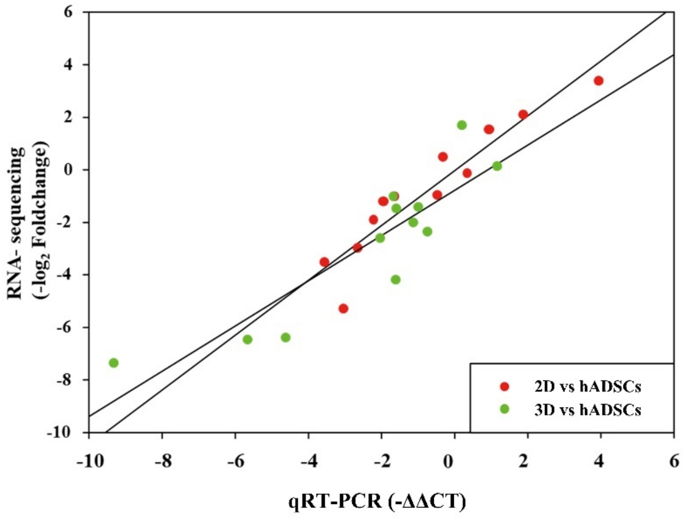

Figure S3. Real-time PCR validation of gene expression levels estimated by RNA-Seq. X-axis: ΔΔCt values from real-time PCR comparing 2D or 3D culture samples and their corresponding controls. Y-axis: log2(fold change) between samples and their corresponding sham controls estimated via RNA-Seq. Red dots represent 2D condition samples and green dots represent 3D condition samples. Pearson's correlation coefficients were 0.85 and 0.95 in 2D and 3D culture conditions, respectively.
